# Intranasal oxytocin administration ameliorates social behavioral deficits in a *POGZ*^WT/Q1038R^ mouse model of autism spectrum disorder

**DOI:** 10.1186/s13041-021-00769-8

**Published:** 2021-03-16

**Authors:** Kohei Kitagawa, Kensuke Matsumura, Masayuki Baba, Momoka Kondo, Tomoya Takemoto, Kazuki Nagayasu, Yukio Ago, Kaoru Seiriki, Atsuko Hayata-Takano, Atsushi Kasai, Kazuhiro Takuma, Ryota Hashimoto, Hitoshi Hashimoto, Takanobu Nakazawa

**Affiliations:** 1 Laboratory of Molecular Neuropharmacology, Graduate School of Pharmaceutical Sciences, Osaka University, Suita, Osaka 565-0871 Japan; 2 Department of Molecular Pharmacology, Graduate School of Pharmaceutical Sciences, Kyoto University, Sakyo-ku, Kyoto, 606-8501 Japan; 3 Department of Cellular and Molecular Pharmacology, Graduate School of Biomedical and Health Sciences, Hiroshima University, Minami-ku, Hiroshima, 734-8553 Japan; 4 Interdisciplinary Program for Biomedical Sciences, Institute for Transdisciplinary Graduate Degree Programs, Osaka University, Suita, Osaka 565-0871 Japan; 5 Molecular Research Center for Children’s Mental Development, United Graduate School of Child Development, Osaka University, Kanazawa University, Hamamatsu University School of Medicine, Chiba University and University of Fukui, Suita, Osaka 565-0871 Japan; 6 Department of Pharmacology, Graduate School of Dentistry, Osaka University, Suita, Osaka 565-0871 Japan; 7 Department of Pathology of Mental Diseases, National Institute of Mental Health, National Center of Neurology and Psychiatry, Kodaira, Tokyo 187-8553 Japan; 8 Osaka University, Suita, Osaka 565-0871 Japan; 9 Division of Bioscience, Institute for Datability Science, Osaka University, Suita, Osaka 565-0871 Japan; 10 Transdimensional Life Imaging Division, Institute for Open and Transdisciplinary Research Initiatives, Osaka University, Suita, Osaka 565-0871 Japan; 11 Department of Molecular Pharmaceutical Science, Graduate School of Medicine, Osaka University, Suita, Osaka 565-0871 Japan; 12 Department of Bioscience, Tokyo University of Agriculture, 1-1-1 Sakuragaoka, Setagaya-ku, Tokyo, 156-8502 Japan

**Keywords:** Social behavior, Autism spectrum disorder, *POGZ*, De novo mutation, Paraventricular nucleus, Oxytocin, Oxytocin receptor, ChIP

## Abstract

Autism spectrum disorder (ASD) is a highly prevalent neurodevelopmental disorder characterized by core symptoms of impaired social behavior and communication. Recent studies have suggested that the oxytocin system, which regulates social behavior in mammals, is potentially involved in ASD. Mouse models of ASD provide a useful system for understanding the associations between an impaired oxytocin system and social behavior deficits. However, limited studies have shown the involvement of the oxytocin system in the behavioral phenotypes in mouse models of ASD. We have previously demonstrated that a mouse model that carries the ASD patient-derived de novo mutation in the *pogo transposable element derived with zinc finger domain* (*POGZ*^WT/Q1038R^ mice), showed ASD-like social behavioral deficits. Here, we have explored whether oxytocin (OXT) administration improves impaired social behavior in *POGZ*^WT/Q1038R^ mice and found that intranasal oxytocin administration effectively restored the impaired social behavior in *POGZ*^WT/Q1038R^ mice. We also found that the expression level of the oxytocin receptor gene (*OXTR*) was low in *POGZ*^WT/Q1038R^ mice. However, we did not detect significant changes in the number of OXT-expressing neurons between the paraventricular nucleus of *POGZ*^WT/Q1038R^ mice and that of WT mice. A chromatin immunoprecipitation assay revealed that POGZ binds to the promoter region of *OXTR* and is involved in the transcriptional regulation of *OXTR*. In summary, our study demonstrate that the pathogenic mutation in the *POGZ*, a high-confidence ASD gene, impairs the oxytocin system and social behavior in mice, providing insights into the development of oxytocin-based therapeutics for ASD.

**Supplementary Information:**

The online version contains supplementary material available at 10.1186/s13041-021-00769-8.

## Introduction

Autism spectrum disorder (ASD) is a neurodevelopmental disorder characterized by core symptoms of impaired social behavior and communication. Its molecular pathology is largely unclear [1]. Although the prevalence rate of ASD is considerable, there are no pharmacological therapeutics for the core symptoms of ASD.

The neuropeptide oxytocin plays a central role in social behavior [2, 3]. Genetic variation in the oxytocin system is associated with social behavior in humans [4]. Recent clinical studies have suggested a potential therapeutic effect of oxytocin in ASD [5]. In mice, targeted disruption of genes encoding oxytocin and its receptor impairs social behavior [6]. Thus, mouse models of ASD provide a useful system for understanding the associations between an impaired oxytocin system and social behavior deficits. However, limited studies have shown the effectiveness of oxytocin in the behavioral phenotypes in mouse models of ASD [7–9].

The *pogo transposable element derived with zinc finger domain* (*POGZ*) is one of the most frequently de novo mutated genes in patients with ASD [10], making POGZ a high-confidence and strong candidate ASD gene (SFARI database). We previously generated a mouse model that carried a pathogenic de novo mutation of *POGZ* identified in a patient with ASD (*POGZ*^WT/Q1038R^ mouse) and found ASD-like behavioral abnormalities in these mice [11], emphasizing the relevance of *POGZ*^WT/Q1038R^ mouse as an ASD model with high construct and face validity. This study explored whether oxytocin administration improves impaired social behavior in *POGZ*^WT/Q1038R^ mice.

## Results

**Fig. 1:**
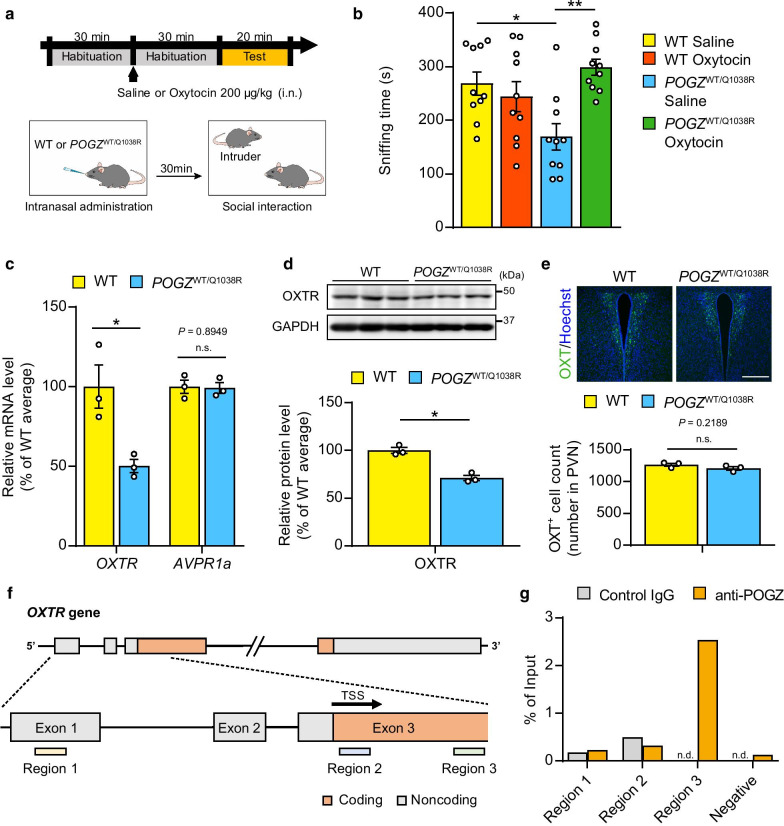
Oxytocin ameliorates impaired social interaction in *POGZ*^WT/Q1038R^ mice. **a** Time course of the reciprocal social interaction test. The test was performed 30 min after intranasal administration of oxytocin (200 μg/kg) or saline. The duration of sniffing was measured during the test. *i.n.* intranasally, *WT* wild-type. **b** Time spent sniffing in the reciprocal social interaction test after oxytocin treatment (each n = 10). **c** qPCR analysis of the expression levels of *OXTR* and *AVPR1a* mRNA in the brain (each n = 3). OXTR, oxytocin receptor; AVPR1a, vasopressin receptor 1A. **d** Representative western blotting and quantification of decreased OXTR in *POGZ*^WT/Q1038R^ mice. GAPDH was used as loading control (each n = 3). Values were normalized to the expression levels of GAPDH. Uncropped western blot images are shown in Additional file 2: Fig. S1. **e** Representative image of OXT (green) and Hoechst 33258 (blue) immunofluorescence in the PVN and stereological counts of OXT-expressing cells in the PVN region of WT and *POGZ*^WT/Q1038R^ mice (each n = 3; scale bar, 300 μm). **f** Schematic diagram of the position of the qPCR amplicons by each primer set in the mouse *OXTR* promoter region. *TSS* translation start site. **g** ChIP assay coupled with qPCR for the quantification of DNA fragments precipitated by an anti-POGZ antibody (n = 1 mice). The results are presented as percentage of the input DNA. *n.d.* not detected. Data are presented as the mean ± SEM. Statistical significance was analyzed by two-way ANOVA, followed by Bonferroni Dunn post hoc tests (**b**, F_1, 36_ = 11.67) and Student’s *t*-test (**c**–**e**). *P < 0.05, **P < 0.01

To examine the effect of oxytocin on social behavior deficits in *POGZ*^WT/Q1038R^ mice, we performed a reciprocal social interaction test 30 min after intranasal administration of saline or 200 μg/kg of oxytocin (Fig. 1a). The dose of oxytocin administration was determined based on the previous report [7, 8]. Consistent with our previous study [11], we confirmed that *POGZ*^WT/Q1038R^ mice treated with saline spent significantly less time sniffing the intruder mice than their WT littermates. We found that intranasal administration of oxytocin successfully ameliorated the decreased sniffing time in *POGZ*^WT/Q1038R^ mice, but it did not significantly affect the sniffing time in WT mice (Fig. 1b).

To characterize the oxytocin system in *POGZ*^WT/Q1038R^ mice, we first measured the expression levels of oxytocin receptor (OXTR) and vasopressin receptor 1A (AVPR1a), which are receptors for oxytocin (OXT) [12]. We found that the OXTR mRNA and protein expression was significantly decreased in *POGZ*^WT/Q1038R^ mice compared to that in WT littermates (Fig. 1c, d). There was no significant difference in the expression levels of *AVPR1a* mRNA between the genotypes (Fig. 1c). We then measured the number of OXT-expressing neurons in the paraventricular nucleus (PVN), a major brain region in OXT synthesis [12]. In contrast to previously reported mouse models of ASD, in which social behavior was curable by OXT [8, 9], we did not detect significant changes in the number of OXT-expressing neurons between the PVN of *POGZ*^WT/Q1038R^ mice and that of WT mice (Fig. 1e).

According to its amino acid sequences and putative domain structures, POGZ is suggested to be involved in transcriptional regulation. Given that the human POGZ-Q1042R (Q1038R in mice) mutation reduces the DNA-binding activity of POGZ [13], we hypothesized that *POGZ*-Q1038R mutation disrupts the binding of POGZ to DNA, resulting in decreased *OXTR* gene expression. To assess whether POGZ binds to the *OXTR* promoter, we performed chromatin immunoprecipitation (ChIP) assays coupled with qPCR (Fig. 1f). Based on a previous study on the *OXTR* promoter [14], we performed qPCR at three sites, upstream (Region 1), directly below (Region 2), and downstream (Region 3) of the translation start site (TSS) in the promoter region of *OXTR*. We found that the DNA sequence containing Region 3 was enriched by anti-POGZ antibody immunoprecipitation, whereas the DNA sequences containing Regions 1 and 2 were not enriched (Fig. 1g). These data suggest that POGZ binds to the *OXTR* promoter downstream of the TSS and regulates *OXTR* gene expression.

## Discussion

A growing body of evidence suggests that oxytocin/vasopressin family peptides are promising therapeutic agents for ASD [4, 5]. However, the possible associations between ASD-associated genetic mutations and the oxytocin system are largely unclear. In this study, we showed that impaired social behavior caused by pathogenic mutation in *POGZ*, a high-confidence ASD gene, can be treated with oxytocin even in the adults. Our study provides insights into the development of oxytocin-based therapeutics for ASD.

Although epigenetic regulation affects the transcription of *OXTR* [12], limited information is available on intracellular and extracellular signals as well as the molecular mechanisms that regulate the transcription of *OXTR*. The downstream pathways of OXTR underlying the regulation of social behavior are also unclear. We found that the pathogenic POGZ mutation identified in a patient with ASD caused decreased *OXTR* expression (Fig. 1c, d). Unlike OXTR-null mice [6], only a slight but significant decrease in OXTR expression is suggested to impair social interaction. Unraveling the precise molecular phenotype of POGZ-mediated regulation of *OXTR* expression will help understand the association between impaired social behavior and OXTR signaling.

Given that histone H3 lysine 9 acetylation and trimethylation, markers for increased and repressed gene expression, respectively, are enriched at the Region 3 in the *OXTR* promoter [14], POGZ binds to the Region 3 and may be involved in the regulation of *OXTR* expression through epigenetic mechanisms. Although our results suggest that POGZ positively regulates *OXTR* expression and that *POGZ*-Q1038R mutation disrupts the transcriptional function of POGZ, recent functional analysis using a luciferase assay showed that POGZ represses transcription [15]. This discrepancy can be reconciled by further investigation of the molecular phenotype underlying the Q1038R mutation.

Our study suggest that pathogenic mutation in one of the high-confidence ASD genes impairs the oxytocin system. Further studies on the possible effects of pathogenic mutations in high-confidence ASD genes on oxytocin signaling are needed to confirm the generalizability of the current findings. Considering the etiological heterogeneity of ASD, focusing on the role of the oxytocin system in ASD can be beneficial for the neurobiological and molecular mechanism-based stratification of patients with ASD, which is important for accelerating therapeutic development.

## Supplementary Information

**Additional file 1.** Detailed materials and methods.

**Additional file 2: Fig. S1.** Raw images of entire membrane of immunoblotting

## Data Availability

Detailed materials and methods are included in Additional file 1. All data supporting the finding of this study are available from the corresponding author on reasonable request.

## Abbreviations

ASD: Autism spectrum disorder
ChIP: Chromatin immunoprecipitation
i.n.: Intranasally
n.d.: Not detected
OXT: Oxytocin
OXTR: Oxytocin receptor
POGZ: Pogo transposable element derived with zinc finger domain
PVN: Paraventricular nucleus
qPCR: Quantitative PCR
TSS: Translation start site
WT: Wild-type
