# Supplementary material for: Intranasal oxytocin administration ameliorates social behavioral deficits in a POGZWT/Q1038R mouse model of autism spectrum disorder
Source: Mol Brain. 2021 Mar 16;14:56. doi: 10.1186/s13041-021-00769-8 (PMC7962304; doi:10.1186/s13041-021-00769-8)
Supplement: Supplementary file 1 — Additional file 1. Detailed materials and methods. [file 13041_2021_769_MOESM1_ESM.pdf]

Additional File 1

Detailed Materials and Methods

Drug administration

Oxytocin (Peptide Institute Inc., Osaka, Japan) was dissolved in 1 mg/mL in saline (Otsuka Pharmaceutical Co., Ltd., Tokushima, Japan) before use. Oxytocin or saline was intranasally administered in mice at a volume of 0.2 mL/kg using a P20 micropipette (Gilson Inc., WI, USA) 30 min before the reciprocal social interaction test. Based on previous reports on mice (Smith et al., Pharmacol. Res., 146:104324 (2019); Galbusera et al., Neuropsychopharmacol., 42:1420-1434 (2017); Neumann et al., Psychoneuroendocrinol., 38:1985-1993 (2013)), the estimated plasma oxytocin concentration in mice in the current study may be virtually similar to that in humans in clinical trials (Striepens et al., Sci. Rep., 3:3440 (2013); Yamasue et al., Mol. Psychiatry, 25:1849-1858 (2020)), however, further experiments are needed to determine the plasma oxytocin concentration in mice used in this study.

Reciprocal social interaction test

Twelve-week-old male WT or *POGZ*$^{WT/Q1038R}$ mice (C57BL/6N genetic background) were used in the test. A test mouse was placed in a test cage and habituated for 60 min. Oxytocin or saline was administered 30 minutes after the beginning of habituation. After habituation, a 7-week-old male mouse (C57BL/N, Japan SLC, Shizuoka, Japan) was placed in the test cage as an intruder mouse. The duration of the sniffing behavior exhibited by the test mouse toward the intruder mouse was measured over the full experimental period (20 min). This test was conducted out between 10:00 and 14:00.

Reverse transcription and real-time PCR

Whole brains from 16-week-old mice were lysed with QIAzol Lysis Reagent (QIAGEN, Hilden, Germany) according to the manufacturer’s instructions. Total RNA from brain lysates was isolated using the PureLink RNA Mini Kit (Thermo Fisher Scientific, MA, USA) according to the manufacturer’s instructions. Total RNA was reverse transcribed using SuperScript III (Life Technologies, CA, USA). Real-time PCR was performed with TB Green Premix Ex Taq II

(Takara Bio Inc., Shiga, Japan) using a CFX96 real-time PCR detection system (Bio-Rad Laboratories, CA, USA). The expression levels of *OXTR* (forward primer sequence: 5′-GTGCAGATGTGGAGCGTCT-3′; reverse primer sequence: 5′-GTTGAGGCTGGCCAAGAG-3′) and *AVPR1a* (forward primer sequence: 5′-CAATTTCGTTTGGACCGATT-3′; reverse primer sequence: 5′-TGTTCAAGGAAGCCAGTAACG-3′) were normalized to those of *GAPDH* (forward primer sequence: 5′-GTGTTCCTACCCCCAATGTG-3′; reverse primer sequence: 5′-TACCAGGAAATGAGCTTGAC-3′) and determined according to the $2^{-\Delta Ct}$ method. The qRT-PCR products from all primer sets revealed a single band at the expected size.

Immunoblotting

Whole brains from 16-week-old mice were lysed using RIPA buffer. Brain lysates were resolved on 7.5% polyacrylamide–SDS gels by SDS–PAGE and transferred to PVDF membranes. Subsequently, the membranes were probed with rabbit anti-OXTR (Abcam, Cambridge, UK, #ab217212, 1:300) and mouse anti-GAPDH (Merck Millipore, MA, USA, #MAB374, 1:5000) antibodies overnight at 4°C, followed by incubation with the secondary antibodies, HRP-conjugated goat anti-mouse IgG (Santa Cruz Biotechnology, CA, USA, #sc-2005, 1:1000) and alkaline phosphatase-conjugated goat anti-rabbit IgG (Santa Cruz Biotechnology, #sc-2007, 1:1000), for 1 h at room temperature. Proteins were visualized via alkaline phosphatase reaction using CDP-star (Roche Life Sciences, Basel, Switzerland) and HRP reaction using Western Lightning Plus ECL (PerkinElmer, MA, USA). Data acquisition and analysis were performed using the LAS4000 image analyzer (GE Healthcare, NJ, USA).

Immunohistochemistry

Ten-week-old mice were deeply anesthetized via an intraperitoneal injection of a mixture of 3 mg/kg medetomidine (Nippon Zenyaku Kogyo, Fukushima, Japan), 4 mg/kg midazolam (Sandoz Pharma, Basel, Switzerland), and 5 mg/kg butorphanol (Meiji Seika Pharma, Tokyo, Japan) and were transcardially perfused with saline, followed by 4% paraformaldehyde (PFA) dissolved in phosphate-buffered saline (PBS). The brains were excised and post-fixed in 4% PFA overnight at 4 °C. The brains were sectioned at a thickness of 30 μm by using a cryostat (Leica, Wetzlar, Germany, CM1520). The brain slices were permeabilized with blocking solution containing 0.3% Triton X-100 (Wako, Osaka, Japan) and 5% bovine serum albumin (Sigma-Aldrich, MO, USA)

in PBS for 1 h and then incubated with the blocking solution combined with rabbit anti-oxytocin-neurophysin 1 (Abcam, #ab2078, 1:1000). The following day, the slices were incubated with the blocking solution combined with Alexa Fluor 488-conjugated goat anti-rabbit IgG (Life Technologies, CA, USA, #A-11008, 1:1000) and Hoechst 33258 dye (Calbiochem, CA, USA) for 1 h. Seventeen to eighteen coronal sections per brain were imaged for quantification of OXT-expressing neurons in the PVN. Images of the stained brain slices were acquired using an Olympus FluoView FV1200 confocal microscope (Olympus, Tokyo, Japan) and a BZ-9000 microscope (Keyence, Osaka, Japan). The images were then analyzed using ImageJ software (NIH, MD, USA).

ChIP assay

ChIP assay was performed as previously described [11]. In brief, chromatin was isolated from the cerebral cortex of WT mice. ChIP was performed with an anti-POGZ antibody (Bethyl Laboratories, TX, USA, A302-509A) and normal rabbit IgG (Merck Millipore, 12-370) using the ChIP-IT Express Enzymatic Magnetic ChIP Kit and Enzymatic Shearing Kit (Active Motif, CA, USA) according to the manufacturer’s instructions. Following ChIP, the DNA samples were purified using the Chromatin IP DNA Purification Kit (Active Motif) and amplified via PCR using the GenoMatrix Whole Genome Amplification Kit (Active Motif). Quantitative PCR (qPCR) was performed using amplified DNA with the previously reported primers [14] for the mouse OXTR promoter region (forward primer for Region 1 sequence: 5′-TGGATTCTCCGCCCAGATTG-3′; reverse primer for Region 1 sequence: 5′-GAGTTTGCAAAGACGCCGAG-3′; forward primer for Region 2 sequence: 5′-AGATGGGAAGACCCCTCCT-3′; reverse primer for Region 2 sequence: 5′-CGCAGCCAACTGGAGTATCG-3′; forward primer for Region 3 sequence: 5′-CAGGGCTGGATGAAGACCG-3’; reverse primer for Region 3 sequence: 5′-AGGTGCACATTTTCTCGCTG-3′). A validated mouse negative control primer set (Active Motif, #71012) was used as a negative control. The enrichment of each amplicon by ChIP was normalized to the amount of amplified DNA fragments immunoprecipitated with the anti-POGZ antibody or normal rabbit IgG.

Statistical analysis

The behavioral data were statistically analyzed using two-way ANOVA, followed by Bonferroni–

Dunn post hoc tests. Quantitative reverse transcription PCR, western blotting and immunohistochemistry were statistically analyzed using Student’s t-test. The significance level was set at $P < 0.05$. Statistical analyses were conducted using Stat-View software (SAS Institute, NC, USA).
