# Supplementary material for: Intranasal oxytocin administration ameliorates social behavioral deficits in a POGZWT/Q1038R mouse model of autism spectrum disorder
Source: Mol Brain. 2021 Mar 16;14:56. doi: 10.1186/s13041-021-00769-8 (PMC7962304; doi:10.1186/s13041-021-00769-8)
Supplement: Supplementary file 2 — Additional file 2: Fig. S1. Raw images of entire membrane of immunoblotting [file 13041_2021_769_MOESM2_ESM.pdf]

Additional File 2

Additional Figure

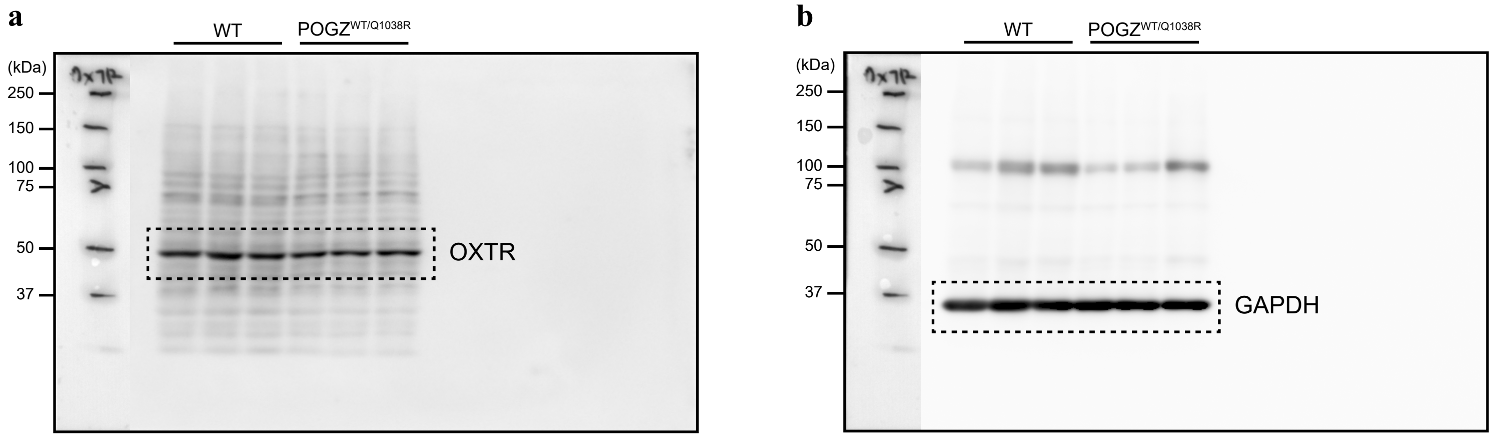

Fig. S1 Raw images of entire membrane of immunoblotting.

a The raw image of immunoblotting of OXTR. **b** The raw image of immunoblotting of GAPDH.
